# Small RNA Sequencing Analysis of miRNA Expression Reveals Novel Insihts into Root Formation under Root Restriction Cultivation in Grapevine (*Vitis vinifera* L.)

**DOI:** 10.3390/ijms21103513

**Published:** 2020-05-15

**Authors:** Hui Li, Zhen Gao, Muhammad Salman Zahid, Dongmei Li, Hafiz Umer Javed, Lei Wang, Shiren Song, Liping Zhao, Wenping Xu, Caixi Zhang, Chao Ma, Shiping Wang

**Affiliations:** 1 Department of Plant Science, School of Agriculture and Biology, Shanghai Jiao Tong University, Shanghai 200240, China; oldhorseli@sjtu.edu.cn (H.L.); salmanzahid2482@hotmail.com (M.S.Z.); LDM651011@sjtu.edu.cn (D.L.); qaziumer@sjtu.edu.cn (H.U.J.); leiwang2016@sjtu.edu.cn (L.W.); sr.song@sjtu.edu.cn (S.S.); lpzhao07@sjtu.edu.cn (L.Z.); wp-xu@sjtu.edu.cn (W.X.); acaizh@sjtu.edu.cn (C.Z.); 2 State Key Laboratory of Crop Biology, College of Horticulture Science and Engineering, Shandong Agricultural University, Tai’an 271018, China; gaoz89@126.com; 3 Institute of Agro-food Science and Technology/Key Laboratory of Agro-products Processing Technology of Shandong, Shandong Academy of Agricultural Sciences, Jinan 250100, China

**Keywords:** microRNA, *Vitis vinifera*, root restriction, root architecture, high-throughput sequencing

## Abstract

Root restriction cultivation (RRC) can influence plant root architecture, but its root phenotypic changes and molecular mechanisms are still unknown. In this study, phenotype observations of grapevine root under RRC and control cultivation (nRC) at 12 time points were conducted, and the root phenotype showed an increase of adventitious and lateral root numbers and root tip degeneration after RRC cultivation from 70 days after planting (DAP). The 70 and 125 DAP sampling of two different cultivations, named nR70, RR70, nR125, and RR125, were selected for small RNA sequencing. A total of 153 known miRNAs and 119 predicted novel miRNAs were obtained. Furthermore, BLAST was used to predict the novel miRNAs with miRBase databases using the default parameters; 96 of the 119 predicted novel miRNAs were similar to other species, and the remaining 23 grapevine-specific novel miRNAs were obtained. There were 26, 33, 26, and 32 miRNAs that were differentially expressed in different comparison groups (RR70 vs. nR70, RR125 vs. nR125, nR125 vs. nR70 and RR125 vs. RR70). Target genes prediction of differentially expressed miRNAs was annotated on a variety of biological processes, and 24 participated in root development. Moreover, multiple miRNAs were found to jointly regulate lateral root development under root restriction conditions. The miRNA expression pattern comparison between RRC and nRC may provide a framework for the future analysis of miRNAs associated with root development in grapevine.

## 1. Introduction

Roots, as an important organ of perennial fruit trees, not only have a mechanical support function for growth, but play a key role in the process of absorbing water and mineral nutrients from the soil [1,2]. Normally, plant roots occur in the radicle of seeds, and root formation can be divided into primary roots, lateral roots, and adventitious roots [3]. The primary root is the first root grown by the plant, usually as the main root; when the main root grows to a certain length, lateral roots emerge from the proximal end of the main root, and continue to emit secondary, tertiary, or multi-level lateral roots; adventitious roots refer to roots emanating from the stems and leaves, which can also emit multi-level lateral roots [4]. Grapevine is a dicotyledonous plant that can be propagated through seeds. During seed propagation, the root system is a taproot system, that is, there are obvious main roots and lateral roots. At the same time, grapevine can also be asexually propagated through cuttings. The root system of the cuttings is formed not by a main root, but by adventitious roots emanating from the stems. The genotypes of offspring obtained by asexual reproduction are the same, and are widely used in scientific research [5,6].

In the early 1990s, researchers were inspired by the practice of garden plant potting and began to explore the cultivation method of limiting the root system of plants, which was called RRC. RRC refers to a cultivation technique that uses physical or ecological materials to control the root system of a plant within a certain volume, and to control the growth space of the root system to regulate the above-ground vegetative and reproductive growth [7]. RRC can significantly inhibit the shoot perimeter, branch length, and leaf area, while improving fruit coloring, increasing the content of pigments such as anthocyanin and carotenoid, increasing the soluble sugar content within the pulp, and reducing the tartaric acid content in grapevine berries [8,9]. These effects may due to the influence of RRC on the absorption of nitrogen and phosphorus in grapevine [10,11]. Our previous studies, in which root morphology observations were conducted, revealed that the root structure was significantly altered under RRC. The dry weight of roots (0.2 cm <thickness <0.5 cm) and fiber roots (<0.2 cm) increased significantly. However, these studies lacked the systematic observation of the whole process of root development. Moreover, there has been little research the molecular mechanisms of grapevine root development, especially under RRC.

Non-coding RNA (ncRNA), which does not encode proteins, is a type of RNA that is widely present in many organisms [12,13], and plays an important role in regulating plant growth and development. Among them, microRNA (miRNA) is a class of endogenous small non-coding RNA with a length of 19–25 nucleotides [14]. MiRNA has been widely reported in various plants and animals and plays a crucial role in post-transcriptional regulation or transcriptional suppression of genes [15]. Plant miRNA not only affects plant resistance to biotic and abiotic stresses, but directly participates in plant growth and development. There have been many miRNAs also reported to influence root development [16,17]. For example, overexpression of ath-miR164 reduced lateral root numbers in *Arabidopsis* [18], and miR160 maintained proper auxin homeostasis and root tip development by regulating *ARF17* in *Arabidopsis* [19,20]. MiR390 influenced lateral root development by regulating the expression of *ARF2*, *ARF3*, and *ARF4* through the secondary siRNA produced by the *TAS3* (trans-acting siRNA 3) gene [21]. Except for these conserved miRNAs, it was reported that some novel miRNAs also participated in root development. For example, miR2111 was found to regulate root node development by long distance transportation [22].

In recent years, high throughput sequencing has been widely used in grapevine to analyze miRNA functions in biotic stress, such as aster yellow phytoplasma infection [23], and abiotic stress, such as cold stress [24]. Moreover, a microRNA expression atlas of grapevine was analyzed, including 70 small RNA libraries containing many tissues [25,26]. In addition, Chitarra et al. integrated a novel miRNA database “miRVIT” inferred from published small RNA libraries that were not uploaded into the miRBase database [27]. However, the miRNA derived from the grapevine root system had less statistical sequencing data. In this study, we analyzed the root phenotype and the expression profile of miRNAs during grapevine root development under both normal and root restriction cultivations, which will provide a framework for the future analysis of miRNAs associated with root development in grapevine.

## 2. Results

### 2.1. Phenotype Variations of Grapevine cv. Muscat Hamburg after Root Restriction Cultivation

The shoot lengths of one-year-old *Vitis vinifera* L. cv. Muscat Hamburg specimens were measured from 60 to 125 days after planting (DAP) under two different cultivations. The results showed that the shoot length of nRC grew faster and reached about 5.5 m, one meter longer than that of RRC (Supplementary Materials Figure S1A). The base diameters of the new shoots in nRC were always higher than those of RRC (Supplementary Materials Figure S1B). The number and length of the secondary shoots were measured between two pruning times, and the length was divided into four grades, denoted I–IV (Supplementary Materials Figure S1C,D). The results showed that the number of secondary shoots in nRC was 18–20 per tree, which was almost two fold greater than the 8–11 per tree of RRCs (Supplementary Materials Figure S1E). Moreover, the length and diameter of the secondary shoots in nRC were significantly higher than those in RRC (Supplementary Materials Figure S1F,G).

Root phenotype photographs were taken at 12 sampling time points (10, 20, 30, 40, 50, 60, 70, 80, 90, 100, 110, and 125 days after planting) from April 24 to August 18, and the nRC and RRC were recorded as nR + DAP (Figure S2) and RR + DAP, respectively (Supplementary Materials Figure S3). The two cultivations showed similar root formation orders including absorbing roots, secondary lateral roots, and new adventitious roots. Finally, the old roots degenerated, and new adventitious roots developed into the main root system. However, after the 70 DAP sampling, the root morphology of the two cultivations changed significantly.

**Figure 1:**
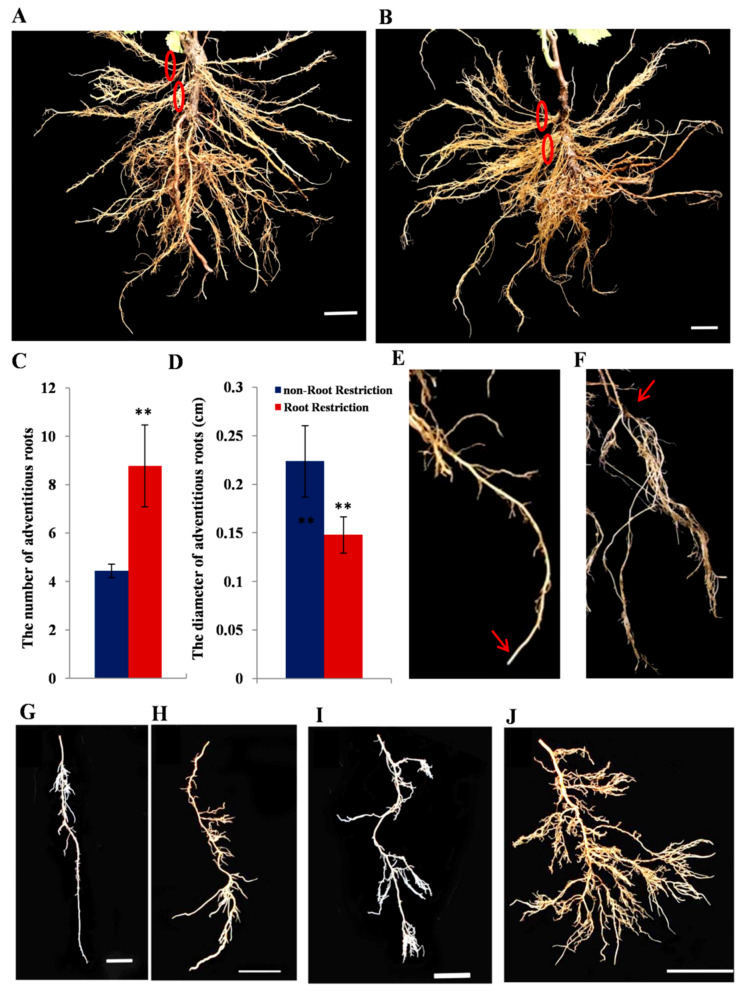
Root phenotype variations of one-year-old grapevine (*Vitis vinifera* L. cv. Muscat Hamburg) after root restriction cultivation. The new adventitious root development in control (**A**) and root restriction cultivation (RRC) (**B**); red circles represent adventitious roots that occurred in clusters. (**C**,**D**) The number and diameter of adventitious roots in two cultivation models; error bars indicate the mean ± SD (n >10) with biological replicates, and values (**) are statistically significant from the control cultivation based on Student’s t test (*P* < 0.01). The root tip in control cultivation (**E**) and RRC (**F**); red arrows represent the initial root tip position; (**G**,**I**) the root morphology in different root positions in control cultivation; (**H**,**J**) the root morphology in the same root position in RRC. Scale bar = 5 cm.

Compared with the nRC, a large number of new adventitious roots with thinner diameters emerged in RRC. Adventitious roots occurred in clusters in both cultivations (Figure 1A,B). The number and diameter of the cluster adventitious roots were analyzed at 70 DAP. The results showed that the number increased significantly after RRC to eight adventitious roots per cluster, which was about twice that of the nRC (four per cluster) (Figure 1C). Furthermore, the diameter of each of the cluster adventitious roots in RRC was about 0.15 cm, which was significantly lower than the nRC (about 0.22 cm) (Figure 1D). The root tip was intact in nRC (Figure 1E), while the root tip growth was defective in RRC, which led to a large number of clustered roots that emerged from the degenerated root tips (Figure 1F). The lateral roots were mainly distributed in the upper part of the roots in nRC (Figure 1G), but in RRC, the lateral roots were densely distributed on the whole roots at 125 DAP (Figure 1H). Occasionally, root tip defects appeared in nRC, and a large number of cluster roots were also emitted from the defective parts (Figure 1I). However, the same type of roots emitted more lateral roots, including secondary or multiple lateral roots, under the RRC (Figure 1J). Root update speed was accelerated after RRC. In the same root, while new roots continued to occur at the lower end, the upper roots began to brown, decay, and disappear (Supplementary Materials Figure S4).

### 2.2. Sequencing Statistics in Different Grapevine Samples

The continuous phenotypic observations of the grapevine root system revealed that there were significant differences between the 70 DAP samplings in RRC and nRC. Next, the 70 and 125 DAP root samples were selected for small RNA sequencing. These four root samples were named as nR70, nR125, RR70, and RR125, and each sample had three replicates, recorded as A, B, and C, respectively. A total of 214,439,588 raw reads were obtained and 168,741,687 clean reads were finally obtained after the quality control steps. The clean reads of each library were between 11.29 and 15.60 M (Table 1). The copy number of FastUniq clean reads ranging from one to ten were more than 96.5%, among which single and double copies accounted for 69.72% and 15.24%, respectively, reaching a total of 85% (Supplementary Materials Figure S5).

### 2.3. Identification of Known and Novel Grapevine miRNAs

**Figure 2:**
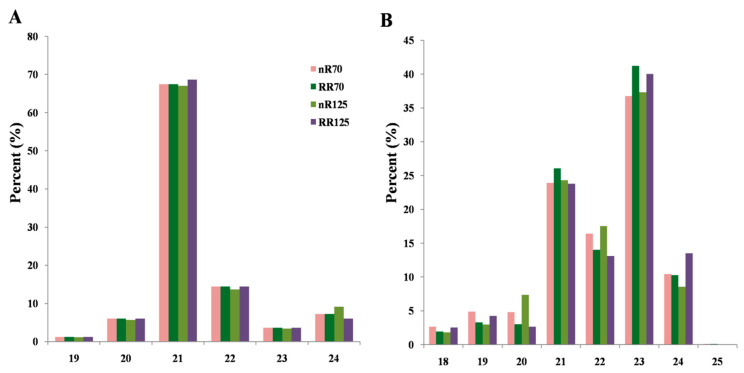
The length distribution percentage of the known (**A**) and novel (**B**) miRNAs in four root sequencing samples. nR70, RR70, nR125 and RR125 represented the 70 days after planting, simplified DAP and 125 DAP sampling time points under control and root restriction cultivation.

After a series of miRNAs prediction analyses, a total of 153 known grapevine miRNAs, and 119 novel miRNAs (named by chromosome random number) were obtained. The length distribution results showed that the known miRNAs were distributed between 19 and 24 nts (nucleotides), of which more than 60% were 21 nts miRNAs (Figure 2A). The length of novel miRNA ranged from 18 to 25 nts, of which the first peak was 23 nts, which accounted for more than 35%, and the proportion was slightly higher in RRC; the second length peak was 21 nts, which accounted for about 25% (Figure 2B). The miRBase database recorded a total of 48 known miRNA families in grapevine, and 45 of them were detected in this study except miR828, miR2950, and miR3628 families. Only 30 miRNA members from 13 known grapevine miRNA families were not detected. The predicted novel miRNAs were searched using the BLAST miRVIT database and 18 of them were perfectly matched. Among them, nine were similar to known miRNA families in grapevine: Un_39994, 6_13658, 19_26046, 19_26048, and 19_25033 were similar to vvi-miR477a; 1_21167 was similar to vvi-miR482; 14_36566 and 17_1792 were similar to vvi-miR3627-5p; and 14_37516 was similar to vvi-miR3633b-3p (Supplementary Materials Table S1).

**Figure 3:**
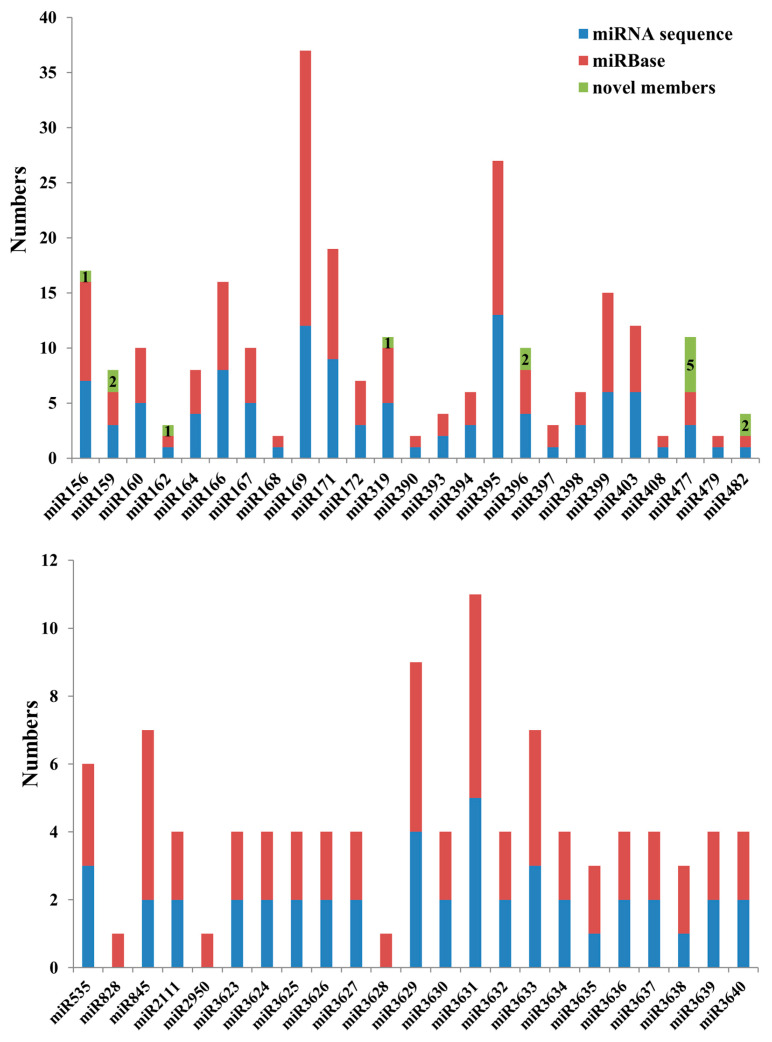
The number of known miRNA family members in grapevine obtained from the miRNA sequence, and the predicted novel miRNA further determined using BLAST with the miRBase.

To obtain the detailed information of the predicted grapevine novel miRNAs, they were aligned with the mature miRNAs database of miRBase using the default parameters. Fourteen conserved grapevine miRNA family members were obtained (Figure 3). Seven of the 14 conserved miRNAs were also detected in the miRVIT database, but they were much better matched in the miRBase than in the miRVIT database. Among them, five novel miRNAs annotated in miRVIT, which were similar to vvi-miR477a, matched better in the miRBase database to ppt-miR477f (*Physcomitrella patens*). Meanwhile, 1_21167 and 14_37516 matched better with mtr-miR482-3p (*Medicago truncatula*) and gma-miR482a -3p (*Glycine max*), respectively. The remaining seven novel miRNAs were 6_12672, 17_2431,11_7793, 9_19848, 15_8904, 2_4979, and 9_20339, which were similar to csi-miR159b-5p (*Citrus sinensis*), mes-miR159a-5p (*Manihot esculenta*), vvi-miR396b, csi-miR156f-3p, ath-miR162a-3p (*Arabidopsis thaliana*), seu-miR319 (*Salicornia europaea*), and osa-miR396e-3p (*Oryza sativa*), respectively (Table 2). In addition, there were 82 novel miRNAs aligned to miRNA families that had been reported in other species but not in grapevine (Supplementary Materials Table S3). The miRNAs 5_32700 and ghr-miR827a (*Gossypium hirsutum*), 14_36566 and vca-miR391-5p (*Vrieseacarinata*), and 14_37655 and mes-miR1446 each had an alignment score of more than 90 points, and mature sequences had high sequence homology with other species (Supplementary Materials Table S2). The remaining 23 novel miRNAs that did not match either database were considered as grapevine-specific novel miRNAs (Supplementary Materials Table S3).

### 2.4. Differentially Expressed miRNAs (DEMs) Analysis

**Figure 4:**
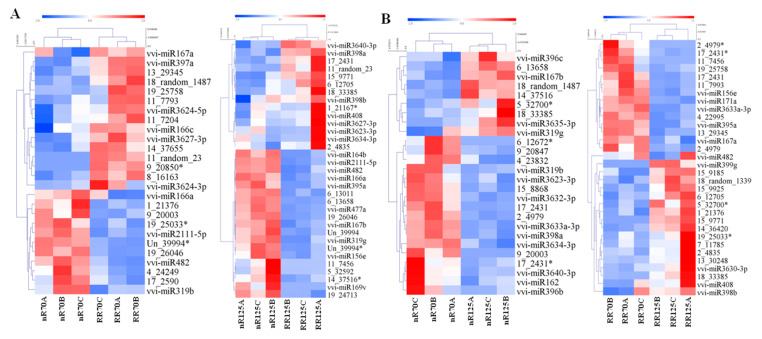
Hierarchical clustering heat map analysis of differentially expressed miRNAs between different cultivations (**A**) and different cultivation stages (**B**) in four root sequencing samples. nR70, RR70, nR125 and RR125 represented the 70 days after planting, simplified DAP and 125 DAP sampling time points under control and root restriction cultivation, A, B, and C represent three replicates.

Principal component analysis (PCA) showed that the distribution was relatively concentrated among biological replicates. Moreover, nR70 and RR70 samples were close and some replicates could not be completely separated, whereas nR125 and RR125 could be completely separated (Supplementary Materials Figure S6). There were 26, 33, 26 and 32 differentially expressed miRNAs (DEMs) identified in different cultivations (RR70 vs. nR70; RR125 vs. nR125) and different cultivation stages (nR125 vs. nR70; RR125 vs. RR70) respectively. Among them, both the known miRNAs and novel miRNAs showed up or down regulation (Supplementary Materials Table S4). Hierarchical clustering heat map analysis showed that different replicates of the same sample clustered together. In different cultivations, both vvi-miR3627-3p and 11_random_23 were up-regulated, while vvi-miR166a, vvi-miR482, vvi-miR2111-5p, Un_39994*, and 19_26046 showed a down-regulated expression (Figure 4A). In different cultivation stages, 5_32700* and 18_33385 were up-regulated, and the down-regulated expressed miRNAs were vvi-miR3633a-3p, 17_2431*, and 2_4979 (Figure 4B). In addition, in the later development stage of the nRC (nR125 vs. nR70), vvi-miR398a, vvi-miR3623-3p, and miR3634-3p were down-regulated, and vvi-miR167b, vvi-miR319g, 6_13658, and 14_37516 were up-regulated, which showed an opposite expression trend of that in RRC (RR125 vs. nR125) (Figure 4B).

**Figure 5:**
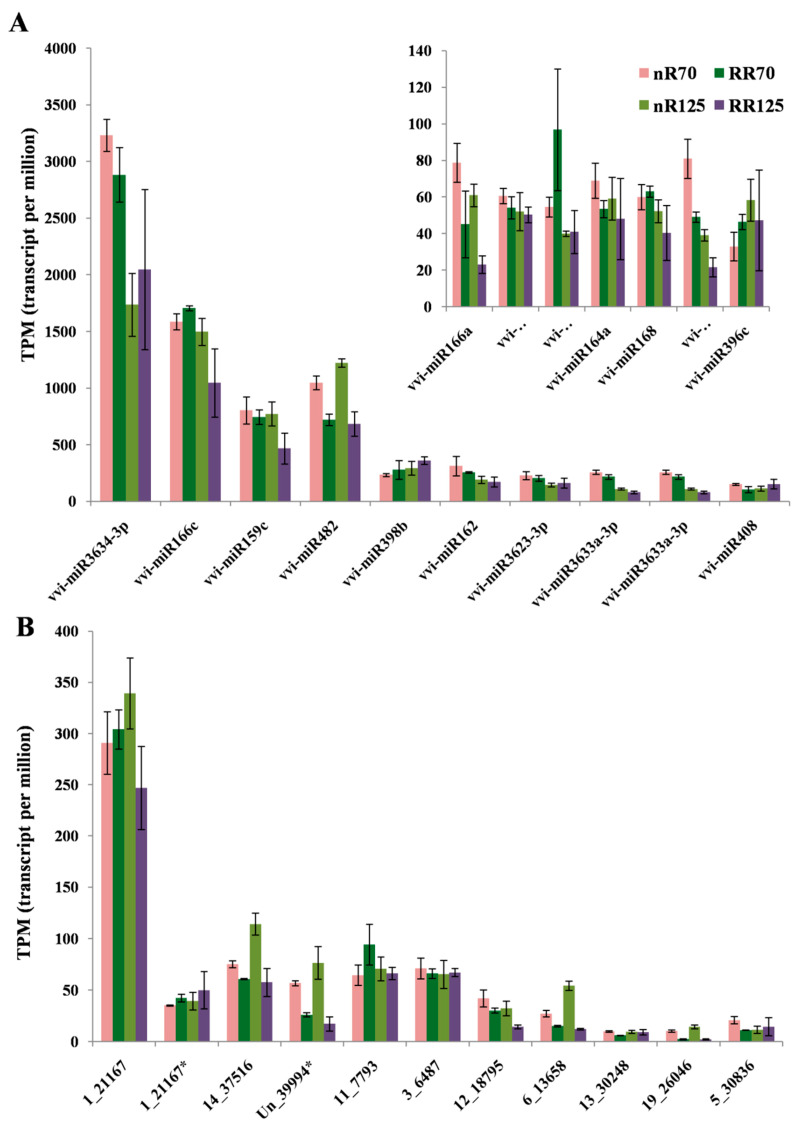
High transcript per million (TPM) values of known miRNAs (**A**) and novel miRNAs (**B**) in four root sequencing samples. nR70, RR70, nR125 and RR125 represented the 70 days after planting, simplified DAP and 125 DAP sampling time points under control and root restriction cultivation.

MiRNA expression level analysis found that known miRNA had higher transcript per million (TPM) values. Seventeen known miRNA had TPM values higher than 30, and the highest value reached was 3000 (Figure 5A). There were only 11 novel miRNAs with TPM values above 10 (Figure 5B). The five known miRNAs with the highest TPM values were vvi-miR3634-3p, vvi-miR166c, vvi-miR159c, vvi-miR482, and vvi-miR398b, and those of novel miRNAs were 1_21167, 1_21167*, 14_37516, Un_39994*, and 11_7793.

### 2.5. Analysis of vvi-miRNA Mediated Grapevine Root Formation

**Figure 6:**
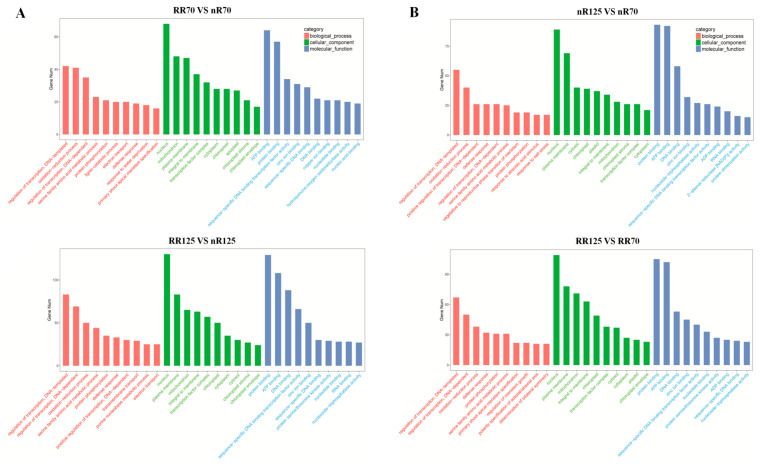
Gene ontology analysis of the predicted target genes of the differentially expressed miRNAs between different cultivations (**A**) and different cultivation stages (**B**) in four root sequencing samples. nR70, RR70, nR125 and RR125 represented the 70 days after planting, simplified DAP and 125 DAP sampling time points under control and root restriction cultivation.

The number of predicted target genes’ mRNAs in DEMs was 344, 738, 402, and 486 in different cultivations (RR70 vs. nR70; RR125 vs. nR125) and different cultivation stages (nR125 vs. nR70; RR125 vs. RR70), respectively. GO annotation analysis revealed that the predicted target genes’ mRNAs participated in a variety of biological processes, and both included regulation of transcription, oxidation–reduction processes, serine family amino acid metabolic processes, and defense response. In different cultivation models, the target genes’ mRNAs had predicted functions in lignin catabolic processes, electron transport, and response to water deprivation (Figure 6A). In addition, response to abscisic acid stimulus, response to salt stress, regulation of meristem growth, and polarity specification of adaxial/abaxial axes were ranked in the top 10 for different cultivation stages (Figure 6B). The first category in cellular component classification was the nucleus, while the protein binding and ATP binding categories were the most abundant categories in molecular function classification. Gene function annotation found a total of 24 target genes’ mRNAs related to root development, which corresponded to 17 vvi-miRNAs. Target genes’ mRNAs of vvi-miR156, vvi-miR166, vvi-miR2111-5p, and vvi-miR3624-3p participated in root hair development, and vvi-miR164 and vvi-miR482 affected lateral root and root cap development. Target genes’ mRNAs of vvi-miR396 were annotated in root development. Target genes’ mRNAs of novel miRNAs had more functions related to root development. For example, the target genes’ mRNAs of 4_24249 and 17_2431 affected primary root development, while those of 15_8868 and 15_8867 participated in root morphogenesis. KEGG analysis was conducted and some target gene mRNAs had corresponding metabolic pathway annotations. Among them, miR2111-5p participated in vasopressin-regulated water re-absorption, corresponding to its GO annotation in root hair development (Table 3).

### 2.6. Vvi-miR160 Family Contributes to Grapevine Root Development

**Figure 7:**
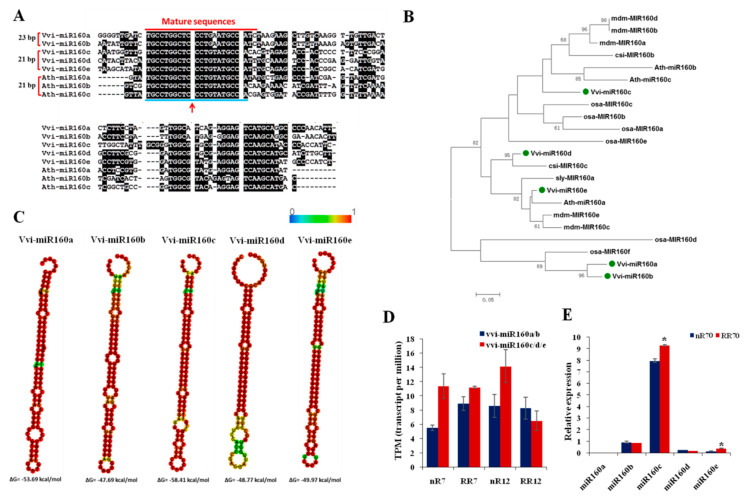
Structural analysis of the vvi-miR160 family. (**A**) Sequence alignment analysis of miRNA precursor between vvi-miR160 and ath-miR160. (**B**) Phylogenetic analysis of miR160 in different species. (**C**) The secondary structure of different vvi-miR160 members. (**D**)Transcript per million (TPM) values ofvvi-miR160 members in four root sequencing samples. (**E**) The relative expression of the precursor of vvi-miR160 members in control and root restriction cultivation at 70 DAP sampling time point; nR70, RR70, nR125 and RR125 represented the 70 days after planting, simplified DAP and 125 DAP sampling time points under control and root restriction cultivation, respectively. Error bars indicate the mean ± SD (n = 3) with biological replicates, and values (*) are statistically significant from the control cultivation based on Student’s t test (*P* < 0.05).

Root tip degradation was one of the most obvious root phenotypes after RRC. MiR160 had been reported to play an important role in root tip development [28]. Five members of the vvi-miR160 family, named vvi-miR160a, b, c, d, and e were obtained using a miRBase search. Among them, the mature sequence lengths of vvi-miR160a and vvi-miR160b were 23 bp, and those of vvi-miR160c, d, and e were 21 bp. There was one base difference between the overlap of vvi-miR160 mature sequences. MiR160 precursor sequence alignment result showed that the flanking sequence was variable, but that the mature sequence was similar. Moreover, the mature sequences of vvi-miR160c, d, and e were 21 bp, which was the same with ath-miR160 (Figure 7A). Phylogenetic analysis of miR160 precursors revealed that vvi-miR160a and vvi-miR160b were clustered into one branch, while vvi-miR160c, d, and e were clustered into another branch, and vvi-miR160c was close to ath-miR160c (Figure 7B). Additionally, RNAfold software was used to predict the stem–loop secondary structures of vvi-miR160 family members according to their precursors, and the vvi-miR160c obtained the highest free energy (Figure 7C). Small RNA sequencing detected vvi-miR160 at a moderate expression level in different sequencing samples, but there was no differential expression among samples (Figure 7D). Quantitative analysis of the relative expression of vvi-miR160 precursors found that vvi-miR160c had the highest expression number, followed by vvi-miR160b, and the expression of vvi-miR160a precursor was not detected (Figure 7E).

## 3. Discussion

Assessing the quality of the small RNA sequencing, there were 186 grapevine miRNAs recorded in the miRBase database, and in this work, 153 of them were detected in grapevine root, with a high detection rate of 82.3%, indicating the crucial role of miRNAs in grapevine root initialization and development (Figure 3). Principal component analysis revealed a close distance between nR70 and RR70 whereas nR125 and RR125 were completely separated (Supplementary Materials Figure S6), which was consistent with the more notable root phenotypic difference in the 12th sampling. Vvi-miR828 had been reported to play a role in the anthocyanin metabolism pathway and to affect fruit coloring [29]. The absence of vvi-miR828 in grapevine root was reasonable, as no anthocyanin accumulation in the root. MiRNA length was considered to be 19–24 nts and usually showed a typical 21 nts and 24 nts two-peak distribution in small RNA sequencing results. In previous research, it was reported that the 24 nts length small RNA was most abundant in grapevine flowers and flower organs (carpels and stamens), and there was no significant peak in miRNA length distribution in seeds [25]. However, the remaining tissues, including roots, had the highest proportion of 21 nts sequences. In this study, the 21 nts sequence had the largest content in known miRNAs (Figure 2A), while novel miRNAs had two peaks at 21 nts and 23 nts in length, while the proportion of 24 nts was relatively small (Figure 2B), which was different from previous studies.

**Figure 8:**
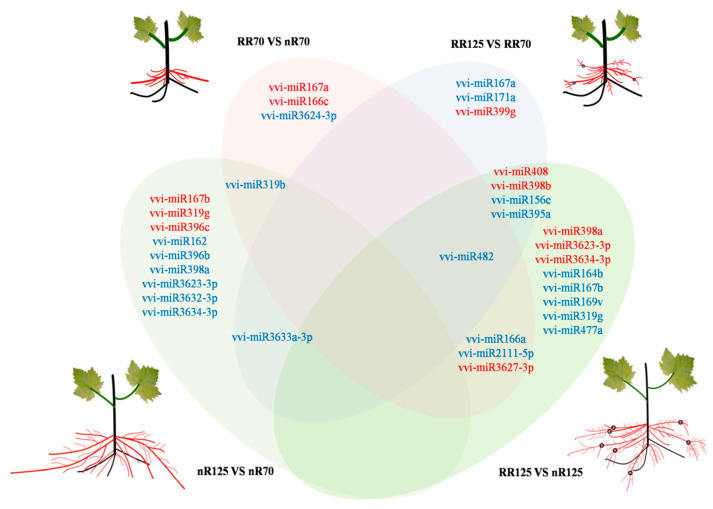
Overview of the root architecture model and miRNAs regulating root formation in grapevine under root restriction cultivation (RRC). The red lines indicate the newly emerging roots, and the black circles indicate the degenerated positions of the root tips after root restriction cultivation; different color ovals represent different sample comparison groups: pink represents RR70 vs. nR70, blue represent RR125 vs. RR70, yellow represents nR125 vs. nR70, green represents RR125 vs. nR125, and the overlay is the intersection of different comparison groups. nR70, RR70, nR125 and RR125 represent the 70 days after planting, simplified DAP and 125 DAP sampling time points under control (nRC) and RRC.

Understanding the root phenotypes of plants under RRC requires further study to determine the molecular mechanisms of root development. Comparing the root system after root restriction cultivation with previous studies, we summarized the characteristics of root development under different cultivation models through continuous observation in grapevine (*Vitis vinifera* L. cv. Muscat Hamburg). Finally, the differences between the two root systems could be attributed to two basic phenotypes, namely the degradation of the root tip and the occurrence of a large number of adventitious roots and lateral roots (Figure 1). The root tip includes the root cap, root meristem, and the root distal region, which showed complex behavioral patterns such as decision-making and played an important role in plant gravitropism. Ath-miR160 has been reported to be involved in root elongation and root cap formation [28]. Overexpression of ath-miR160c displayed uncontrolled cell division in the root distal region and loss of gravity-sensing. Moreover, the root length of the seedling was reduced, the lateral root number was increased, and its target gene’s *arf10arf16* double mutant showed the same phenotype. Phylogenetic analysis revealed that vvi-miR160c was in the same clade as ath-miR160c (Figure 7), which indicated vvi-miR160c may influence root tip development in grapevine. In addition, multiple miRNAs have been reported to participate in lateral root development in plants (Figure 8). For example, miR156 targeted *Spls* [30], miR164 targeted *NAC* [18], miR396 targeted *bHLH74* [31], and miR171 targeted *GRAS* [32] to regulate lateral root development. These miRNAs involved in lateral root development were differentially expressed in at least one of the root development stages in grapevine. Among them, miR167 was reported to negatively regulate the numbers of lateral roots. In this study, miR167a was up-regulated, while miR167b was down-regulated after RRC, indicating that functional differences exist among miRNA family members. The down-regulated expression of miR167b is in line with the characteristics of promoting lateral root development, which deserves further study. Adventitious roots are an indispensable feature of large-scale plant reproduction. MiRNAs affected the development of adventitious roots by targeting *auxin response factors* [33]. In *Arabidopsis*, ath-miR160 can target auxin response factor *AtARF17* to regulate the occurrence of adventitious roots, in which the number of adventitious roots increased after overexpression of ath-miR160 in *Arabidopsis*, while the number of adventitious roots decreased in plants overexpressing *AtARF17.AtARF6* and *AtARF8* are positive regulators that promote adventitious root growth, and both are strictly regulated by ath-miR167. Moreover, it was found that *AtARF6*, *AtARF8*, and *AtARF17* were functionally redundant and had an additive effect in regulating the occurrence of adventitious roots. Overexpression of *AtARF17* in the background of *arf6arf8* mutants had the least number of adventitious roots. Combined with ath-miR160 targeted *AtARF10* and *AtARF16* that affected root tip development, miR160 was indispensable for the overall development of the root system. Although vvi-miR160c showed no difference in expression of root development after RRC, the role of vvi-miR160 is still worthy of further study.

The root system explores the soil for nutrients. In this exploration process, due to space limitations, the relative soil amount of the root system decreased. Therefore, in the case of insufficient soil, the root system may supplement the deficiency of root tip degradation by issuing more lateral roots, which increases the root surface area and helps in the search for more soil and nutrients for growth. With continuous growth of the root system, the roots become thinner. Root restriction cultivation is the spatial stress response to growth of the root system. In this study, some differentially expressed miRNAs after RRC were rarely reported to participate in root development, but played a role in biotic or abiotic stresses (Figure 8). Among them, miR482 was a miRNA related to disease resistance [34,35], which is a highly conserved miRNA and only found in some species. MiR2111 was detected in the phloem sap under phosphorus-limited conditions, and the abundance of miR2111 in the phloem sap of *Brassica napus* strongly depended on the concentration of P or N, suggesting that it was effective at low phosphorus in plant roots [36]. In addition, miR167 targeted *IAR3* [37], miR169 targeted *NFYAs* [38], miR398 targeted *CSDS* [39], and miR408 [40] was involved in drought stress. All these results indicate that RRC is part of a combination of multiple stress processes, and the effect of drought was obvious. Meanwhile, several newly discovered miRNA family members, namely miR3623-3p, miR3627-3p, miR3632-3p, miR3633a-3p, and miR3634-3p, were also detected and were also highly and differentially expressed, but their functions are still unclear and needed further study.

## 4. Materials and Methods

### 4.1. Plant Materials

The grapevine materials used in this study were cuttings. One-year-old *Vitis vinifera* L. cv. Muscat Hamburg with roots that formed in the autumn of 2016 were planted in the greenhouse of the Fruit Tree Laboratory in Shanghai Jiao Tong University (31°11′ N, 121°29′ W) in 2017. Two different cultivation models including Root restriction cultivation (referred to RRC) and control cultivation (referred to nRC) were used in this study. In root restriction cultivation, 100 cuttings were cultivated in the root zone container with a diameter of 30 cm and a height of 30 cm (with holes around it) and separated from the ground by a tray. The planting substrate was soil, organic fertilizer and perlite with 1:1:1 mixed. In control cultivation, 100 plants were planted on the ground with a height of 40 cm in the same substrate. The initial planting distance was 70 cm × 70 cm. Sampling was performed in a zigzag pattern to ensure that there was sufficient space for root development. The above-ground management were the same, and all of them maintain single-vine growth with no topping. The secondary shoots were trimmed every 7_10 days. Moreover, the experimental materials were equipped with unified control irrigation measures. The roots firstly sampled on April 24, 2017 when above-ground started to sprout, and then roots were sampled at 10-day intervals during the first 11 time points and at 15-day intervals at the last time point. Finally, 24 grapevine root samples from two different cultivation models at 12 time points (10, 20, 30, 40, 50, 60, 70, 80, 90, 100, 110, and 125 days after planting) were collected. At each sampling time point, 6_9 trees were selected as biological replicates.

### 4.2. Small RNA Libraries Construction and Illumina Sequencing

According to root morphology observation, small RNA sequencing was performed on four root samples from the 70 DAP and 125 DAP sampling time points under two cultivation conditions. Total RNA was extracted from collected root samples using CTAB method. RNA concentration and quality were detected using both NanoDrop 2000 (Thermo Scientific, Waltham, MA, USA) and Agilent 2100 Bioanalyzer (Agilent Technologies, Palo Alto, CA, USA). 12 small RNA Libraries (four samples each with three biological replicates) were constructed according to the TruSeq Small RNA Sample Preparation Guide kit (Illumina, Foster City, CA, USA) and sequenced using Illumina Hiseq 2000. Briefly, 1 μg of total RNA was ligated to the 3’- and 5’-sequencing adapters by T4 RNA ligase and reversed into cDNA by Super Script III reverse transcriptase (Invitrogen, New York, NY, USA). The obtained cDNA template was PCR amplified using adaptor primers for 15 cycles. The product was separated and purified by 6% Novex TBE polyacrylamide gel electrophoresis (Invitrogen, New York, NY, USA), and RNA fragments in the range of 147–157 nts were excised and recovered. The length and quality of the library were determined by Agilent 2100 bioanalyzer. The RNA-Sequencing raw data have been deposited to the National Centre for Biotechnology Information (NCBI) BioProject database under accession number PRJNA601829. All the supporting data are included as additional files.

### 4.3. Identification of Known and Novel vvi-miRNAs

The criteria for the raw data quality control included: (1) remove the adapter sequence by cutadapt [41] software, and filter sequences less than 15 bp and greater than 41 bp in length; (2) use fastx_toolkit [42] software to perform Q20 quality control and retain sequences with Q20 above 80%; (3) filter reads containing N bases by NGSQCToolkit [43] and get clean reads; (4) remove redundant sequence and obtained clean reads uniq.

The process to obtain known and novel vvi-miRNAs contained the following steps: (1) Clean reads were mapped to the reference *V. vinifera* L. cv. Pinot Noir (PN40024) genomes to remove unmapped reads. (2) compared the filtered reads with Rfam [44] (version 10.0) database by blastn [45], extracted the results with E-value ≤ 0.01, annotates and removed the sequences such as rRNA, snRNA, snoRNA, tRNA; (3) remove sequences perfect matched with the transcript and longer than 26 bp and less than 15 bp by bowtie [46] software; (4) remove redundant sequence by RepeatMasker [47] software. After the filtration and removal steps, the remaining sequences were perfect matched with mature miRNAs database in the miRBase [48], Sequences with perfect matches were considered as known miRNAs in *V.vinifera*. The unannotated sRNAs were performed secondary structure prediction by RNAfold [49] database, and sequences with miRNA hairpins were considered as novel miRNAs.

Moreover, the predicted novel miRNAs were searched in the miRVIT [27] database to annotate novel miRNAs that had been reported in grapevine before. In addition, the novel miRNA also aligned with mature miRNAs database in the miRBase 21 by the default parameter (Evalue cutoff ≤ 10, Mismatch penalty = −4, Match score ≥ 60). Aligned novel miRNAs were considered as non-conserved miRNAs, while unaligned ones were considered as grapevine specific novel miRNAs.

### 4.4. Differentially Expressed miRNAs Analysis and Annotation of the Target Genes

MiRNA expression quantification was normalized according to the expression of transcript per million (TPM) [50] and calculated as: TPM = Reads count of per miRNA/Reads count of per miRNA × 10^6^. DESeq [51] (v1.18.0) software in the R package was used for miRNA differential expression analysis, the p value was calculated, and miRNAs with a *P* value < 0.05 were selected. At the same time, hierarchical clustering analysis using the Euclidean distance measurement with MeV software was performed on differentially expressed miRNAs between different samples. Target genes were predicted using targetfinder [52] software, and GO functional annotation and KEGG analysis were performed.

### 4.5. Structure Analysis of vvi-miR160 Family

The miR160 family members and sequence information of grapevine and *Arabidopsis* were retrieved by NCBI (www.ncbi.nlm.nih.gov), and the obtained sequences were aligned and analyzed by BioEdit [53] software. In addition, vvi-miR160 was blast in NCBI to find miR160 sequences with high homology to other species. MEGA 6 [54] software was used for phylogenetic analysis of miR160 in different species. The RNAfold [49] was used to predict the secondary structure of different vvi-miR160 members. Gene accession number listed in Supplementary Materials Table S5

### 4.6. RT-qPCR Analysis of the Expression Levels of vvi-miR160 Family

RNA extraction of grapevine root was performed according to the instructions of TianGen RNAprep Pure Plant Kit (TIANGEN, Beijing, China, DP441). RNA quality was detected by 1.2% agarose gel electrophoresis and a NanoDrop 2000 system (Thermo Scientific, Waltham, MA, USA) to ensure RNA OD 260/280 values ranged from 1.8 to 2.0. First-strand cDNA was generated according to the steps of PrimeScript™ RT Master Mix (TaKaRa, Dalian, China, RR036A). Primer Premier 5 software was used for gene-specific primer design (Supplementary Materials Table S5). The RT-qPCR was performed on a CFX Connect Real-Time Detection System (Bio Rad, Hercules, USA). A 10-μL reaction volume included 5 μL of TB Green Premix Ex Taq II (TAKARA, Dalian, China), 0.5 μL of each primer (10 μM), 1 μL of cDNA template (diluted 10-fold), and 3 μL of double-distilled water. Two-step RT-qPCR was performed using the following conditions: initial denaturation at 95 °C for 150 s, followed by 40 cycles at 95 °C for 5 s and 60 °C for 30 s. The *VvGAPDH* (XM_002263109.3) was used as an internal control [55]. The relative expression of the genes was calculated using the 2^−ΔΔCt^ method. Each experiment was repeated three times.

### 4.7. Statistical Analyses

Statistical significance was evaluated by Student’s t test analysis.

## 5. Conclusions

Grapevine root architecture had been changed in root restriction cultivation after planting for 70 days, which was mainly manifested as root tip degradation, subsequently caused a large number of lateral roots, and also enhanced the rate of root regeneration. Small RNA sequencing was performed on the 70 DAP and 125 DAP sampling time points of the root restriction cultivation and control. A total of 153 known miRNAs and 119 predicted novel miRNAs were obtained. Annotations of the novel miRNAs by miRVIT and miRbase database obtained 14 known new miRNA members and 23 grapevine-specific miRNAs. Differentially expressed miRNAs analysis found that multiple miRNAs were reported to be involved in root system development, and biotic and abiotic stresses, indicating that root restriction cultivation was jointly regulated by multiple miRNAs, and multiple minor stresses exist in root development on root restriction condition. In addition, the specific expression of vvi-miR160c in the apex may be the main cause of apical degradation, which leads to the phenotype of root restriction cultivation.

## Figures and Tables

**Table 1:** The statistical information of miRNA in high throughput sequencing.

Sample	Raw Reads	Reads Trimmed Length	Reads Trimmed_Q20	Reads Trimmed_N	Clean Reads	Clean Reads Uniq
nR70A	17,440,227	13,828,456	13,805,784	13,791,775	13,791,775	2,789,195
nR70B	16,785,530	12,712,507	12,694,953	12,682,543	12,682,543	2,424,073
nR70C	16,705,937	12,741,508	12,719,806	12,706,889	12,706,889	2,449,057
RR70A	19,603,314	16,149,312	16,130,501	16,129,531	16,129,531	3,896,588
RR70B	16,454,204	13,692,986	13,676,330	13,662,899	13,662,899	3,129,670
RR70C	14,509,081	11,845,746	11,840,120	11,839,622	11,839,622	2,768,680
nR125A	17,921,125	14,407,233	14,391,593	14,377,909	14,377,909	3,583,714
nR125B	18,875,954	14,074,226	14,050,637	14,036,500	14,036,500	3,639,479
nR125C	17,047,738	12,015,900	11,997,503	11,985,776	11,985,776	3,224,084
RR125A	19,152,378	15,408,723	15,401,439	15,400,735	15,400,735	3,942,147
RR125B	20,246,983	16,368,921	16,361,306	16,360,589	16,360,589	4,167,784
RR125C	19,697,117	15,784,039	15,767,917	15,766,919	15,766,919	4,360,497
Total	214,439,588	169,029,557	168,837,889	168,741,687	168,741,687	40,374,968

**Table 2:** Predicted new known miRNA member information after alignment with mature sequence in miRBase with default parameters.

Provisional ID	Mature Sequence	Length	Loc_miRNA	miRBase Alignment	Query Alignment	Subject Alignment	Strand	Score	Evalue
6_12672	AGCUGCCGACUCAUUCAUUCA	21	chr6:9137310_9137389:+	csi-miR159b-5p	1_21	1_21	+	105	0.002
17_2431	AGCUGCUGACUUAUGGAUCCC	21	chr17:2609257_2609342:-	mes-miR159a-5p	1_21	1_21	+	87	0.065
9_19848	CUCUCUGCUACCGUCAUUCUGC	22	chr9:19106874_19106939:+	csi-miR156f-3p	1_18	4_21	+	63	7
15_8904	UCGAGAAACCUCUGCAUCA	19	chr15:9049770_9049811:+	ath-miR162a-3p	1_18	1_18	+	81	0.2
2_4979	AUUGAAUGAUGCGGGAGACA	20	chr2:855616_855687:-	seu-miR319	3_20	3_20	+	81	0.22
11_7793	UCCCACAGCUUUCUUGAACUU	21	chr11:5246803_5246886:-	vvi-miR396b	1_20	1_20	+	91	0.03
9_20339	CUUUCUUGAACCAAUGGGUCCCAUU	25	chr9:5984685_5984768:-	osa-miR396e-3p	1_13	2_14	-	65	4.3
19_26046	GUUGGAAGUCGGUGGGGGACC	21	chr19:18872730_18872799:-	ppt-miR477f	1_18	1_18	+	90	0.037
19_25033	UCCCUCAAAGGCUUCCAAUUU	21	chr19:18678362_18678430:+	ppt-miR477f	1_19	1_18	+	90	0.037
6_13658	CUGGAAGCCGAUGGGGGACC	20	chr6:19950754_19950822:-	ppt-miR477f	1_20	1_18	+	90	0.037
Un_39994	UCCCUCAAAGGCUUCCAAUUUU	22	chrUn:16672987_16673055:-	ppt-miR477f	1_21	1_18	+	90	0.04
19_26048	AAGUUGGAAGCCGGUGGGGGA	21	chr19:18881377_18881443:-	ppt-miR477f	3_21	1_19	-	68	2.5
14_37516	UUCCCAAUGCCGCCCAUUCCAA	22	chr14:19755490_19755563:-	gma-miR482a-3p	1_22	3_24	+	92	0.027
1_21167	UCUUACCAACACCUCCCAUUCCA	23	chr1:3865584_3865662:+	mtr-miR482-3p	1_22	1_21	+	87	0.065

**Table 3:** Predicted target genes’ mRNA of differentially expressed miRNAs related to root development.

MiRNA_id	Target Gene’ mRNA	Target Start	Target End	Strand	Score	Target Seq (5′->3′)	Query Seq (3′->5′)	GO id	Gene Function Annoation	KEGG Pathway	KEGG Pathway Description
4_24249	VIT_214s0068g01330	3178	3196	+	4	AAACGGAAGAACCACCAUA	AUAGCCUUAUUGGUGGUAU	GO:0080022	primary root development	NA	NA
17_2431	VIT_212s0057g00680	1326	1346	+	4	GGAGUUCCUGAGUCAGCAGCU	CCCUAGGUAUUCAGUCGUCGA	GO:0080022; GO:0010071; GO:0010078	primary root development |root meristem specification | maintenance of root meristem identity	NA	NA
15_8868 > 15_8867	VIT_208s0058g01340	2392	2409	+	3	CAUGCUGCUGCCAGCCCA	CCACCACGACGGUCGGGU	GO:0016021	root morphogenesis	NA	NA
15_8868 > 15_8867	VIT_211s0037g00040	1169	1185	+	4	GGUGGUGCU-CCAGCACA	CCACCACGACGGUCGGGU	GO:0080147	root hair cell development	NA	NA
6_12672*	VIT_203s0063g02440	545	562	+	4	ACC-CCCGUAUUAUUCAUA	UGGAGGGCGUAGUAAGUGU	GO:0048364	root development	NA	NA
6_13011	VIT_211s0016g01900	119	136	+	4	AAGUCAUCGCA-CACAGCA	UUCAGUAGAGUAGUGUCGU	GO:0048528	post-embryonic root development	NA	NA
9_20003	VIT_214s0066g00370	1930	1947	+	4	GCUGUCUUG-CCACCAGAG	UGGCGGAACUGGUGGUCUU	GO:0010053	root epidermal cell differentiation	NA	NA
9_20003	VIT_214s0066g00370	1930	1947	+	4	GCUGUCUUG-CCACCAGAG	UGGCGGAACUGGUGGUCUU	GO:0010053	root epidermal cell differentiation	NA	NA
19_25758	VIT_201s0010g03300	3037	3054	+	4	GAGGCAUCCGUAUUUUCA	CUCGGUAGGCGUCAAAGU	GO:0010053	root epidermal cell differentiation	ko00510, ko04141	N-Glycan biosynthesis | Protein processing in endoplasmic reticulum
19_24713	VIT_213s0019g02390	1712	1728	+	4	UCAUUCA-CUCAAAAUCU	AGUCAGUUGGGUUUUAGA	GO:0048767; GO:0010449	root hair elongation | root meristem growth	ko00790	Folate biosynthesis
19_24713	VIT_213s0019g04320	129	146	+	4	UUGUUCAACCCCAAAUCU	AGUCAGUUGGGUUUUAGA	GO:0048765	root hair cell differentiation	NA	NA
19_24713	VIT_203s0038g03620	4817	4833	+	4	UCAG-CAACCAAAAAUCA	AGUCAGUUGGGUUUUAGA	GO:0048767	root hair elongation	NA	NA
vvi-miR3624-3p	VIT_205s0029g00330	1110	1130	+	4	AGGGCUGCGCUGCUGCCCUGA	UCAUCAUACGACGACGGGACU	GO:0005829	root hair elongation	ko00270	Cysteine and methionine metabolism
2_4979*	VIT_218s0001g04980	889	907	+	4	GGAUGAAUGAGUCGG-AGAU	CCUACUUACUCAGCCGUCGA	GO:0048364	root development	ko01212, ko00620, ko00640, ko00061, ko00254, ko04152	Fatty acid metabolism | Pyruvate metabolism | Propanoate metabolism | Fatty acid biosynthesis | Aflatoxin biosynthesis | AMPK signaling pathway
2_4979*	VIT_207s0289g00100	395	413	+	4	GGAUGAAUGAGUCGG-AGAU	CCUACUUACUCAGCCGUCGA	GO:0048364	root development	NA	NA
vvi-miR396	VIT_208s0007g03760	703	723	+	3	CGUUCAAGAAAGCCUGUGGAA	UCAAGUUCUUUCG-ACACCUU	GO:0048364	root development	NA	NA
vvi-miR2111-5p	VIT_208s0007g01270	1635	1655	+	0	UAGACCUCAGGAUGCAGAUUA	AUCUGGAGUCCUACGUCUAAU	GO:0080147	root hair cell development	ko04962	Vasopressin-regulated water re-absorption
vvi-miR156	VIT_204s0008g00960	2493	2512	+	1	GUGCUCACUCUCUUCUGUCA	CACGAGUGAGAGGAGACAGU	GO:0048767	root hair elongation	ko04010, ko04020	MAPK signaling pathway | Calcium signaling pathway
vvi-miR166	VIT_209s0002g03740	1510	1528	+	0.5	GGAAUGAAGCCUGGUCCGG	CCUUACUUCGGACCAGGCU	GO:0048765	root hair cell differentiation	NA	NA
vvi-miR166	VIT_206s0004g02800	1730	1748	+	1	GGGAUGAAGCCUGGUCCGG	CCUUACUUCGGACCAGGCU	GO:0045595	root hair cell differentiation	NA	NA
vvi-miR166	VIT_213s0019g04320	1114	1132	+	1	GGGAUGAAGCCUGGUCCGG	CCUUACUUCGGACCAGGCU	GO:0008289	root hair cell differentiation	NA	NA
vvi-miR166	VIT_209s0002g03740	1508	1528	+	2.5	CUGGAAUGAAGCCUGGUCCGG	CCCCUUACUUCGGACCAGGCU	GO:0048263	root hair cell differentiation	NA	NA
vvi-miR164	VIT_219s0027g00230	589	609	+	1.5	AGCAAGUGCCCUGCUUCUCCG	UCGUACACGGGACGAAGAGGU	GO:0048527	lateral root development	NA	NA
vvi-miR482	VIT_218s0001g03540	372	392	+	4	GGAGUGAGAGGAG-AGGAAAGG	CCUUACCCUCCUCAUCCUUUCU	GO:0010311; GO:0048829	lateral root formation | root cap development	ko04075	Plant hormone signal transduction

## Supplementary Materials

Supplementary materials can be found at https://www.mdpi.com/1422-0067/21/10/3513/s1. **Figure S1.** Aboveground phenotype variations of one-year old grapevine cv. Muscat Hamburg after root restriction cultivation. The length (A) and diameter (B) of shoots in control and root restriction cultivations grew from 60 to 125 days after planting. The grading of secondary shoots in root restriction cultivation (C) and control (D) according to their length. (E) The number of secondary shoots per tree between two pruning. (F) and (G) represent the length and diameter of secondary shoots in different length grades. Error bars indicate the mean ± SD (n ≥ 10) with biological replicates, and values (*) are statistically significant from the control cultivation based on Student’s t test (*P* < 0.05). **Figure S2.** The root morphology of one-year old grapevine cv. Muscat Hamburg in control cultivation at 12 sampling time points (10, 20, 30, 40, 50, 60, 70, 80, 90, 100, 110, and 125 days after planting), recorded as nR + DAP. **Figure S3.** The root morphology of one-year old grapevine cv. Muscat Hamburg in Root restriction cultivation at 12 sampling time points(10, 20, 30, 40, 50, 60, 70, 80, 90, 100, 110, and 125 days after planting), recorded as nR + DAP. **Figure S4**. The root updated morphology after root restriction cultivation. White arrows indicate the position of root begun to brown, decay and disappear. Bar = 5 cm. **Figure S5.** The copy number of read counts in the high throughput sequencing. **Figure S6**. The principal component analysis of different sequence samples in grapevine. nR70, RR70, nR125 and RR125 represented the 70 days after planting, simplified DAP and 125 DAP sampling time points under control and root restriction cultivation, and each sample had three replicates, recorded as A, B, and C, respectively. **Table S1** The information of the predicted novel miRNAs aligned with miRVIT database. **Table S2** The information of predicted unconserved grapevine miRNA after alignment with mature sequence in miRBase with default parameters. **Table S3** The information of predicted grapevine specific miRNA. **Table S4** The information of differentially expressed miRNAs between different cultivation and different cultivation stages. **Table S5** The primers and accession number of genes used in this study.

## Abbreviations

ARF	Auxin response factor
bHLH	basic helix-loop-helix
DAP	Days after planting
DEMs	Differentially expressed miRNAs
miRNA	microRNA
NCBI	the National Centre for Biotechnology Information
ncRNA	Non-coding RNA
nRC	Non-Root restriction cultivation
PCA	Principal component analysis
RRC	Root restriction cultivation
Spl	Squamosa-promoter binding protein-like
TAS	Trans-acting siRNA genes
TPM	Transcript per million
