# Supplementary figures and images for: Small RNA Sequencing Analysis of miRNA Expression Reveals Novel Insihts into Root Formation under Root Restriction Cultivation in Grapevine (Vitis vinifera L.)
Source: Int J Mol Sci. 2020 May 15;21(10):3513. doi: 10.3390/ijms21103513 (PMC7278995; doi:10.3390/ijms21103513)

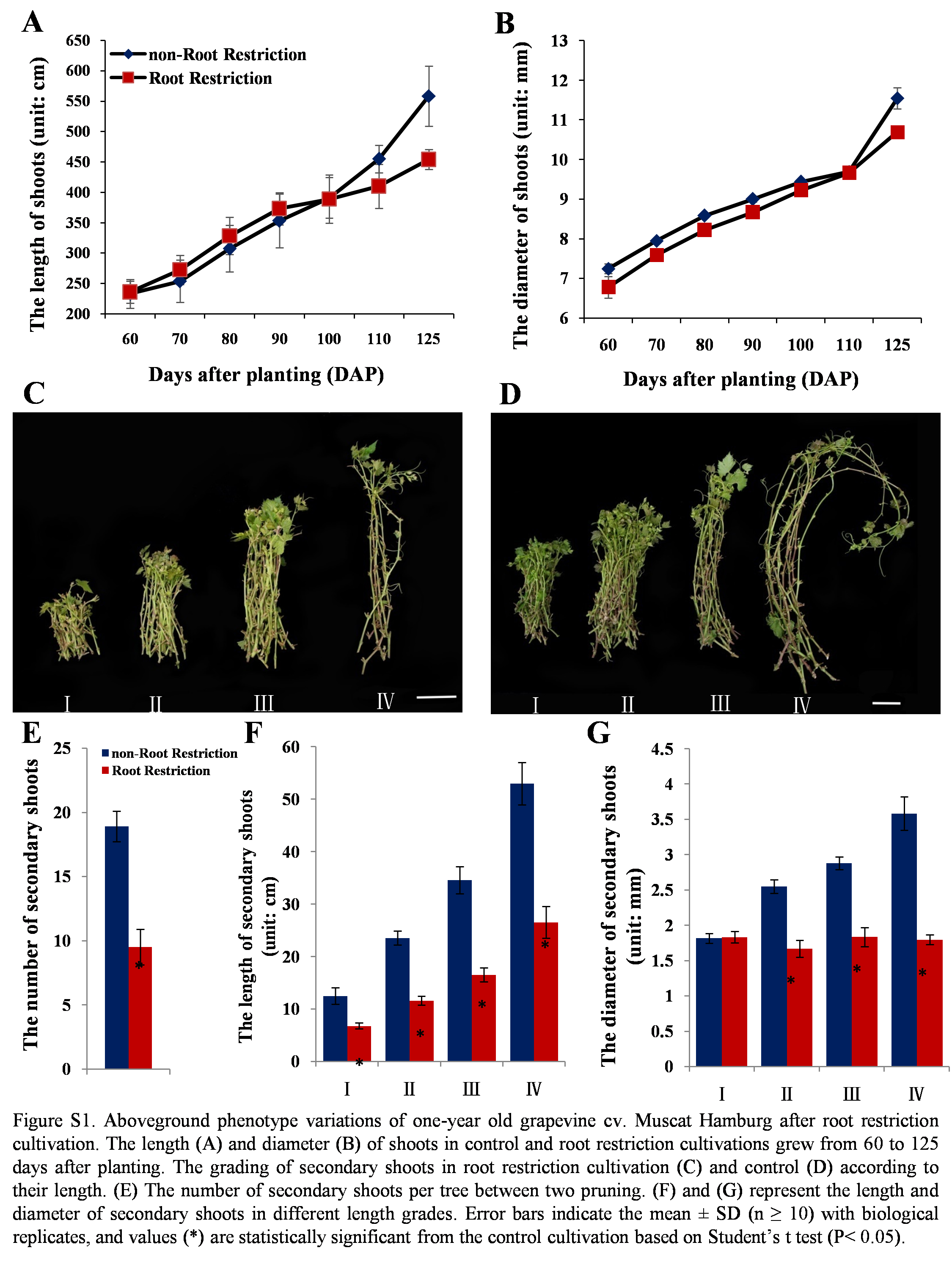

Supplement: Supplementary file 1 [file ijms-21-03513-s001.zip › Figure S1.tif]

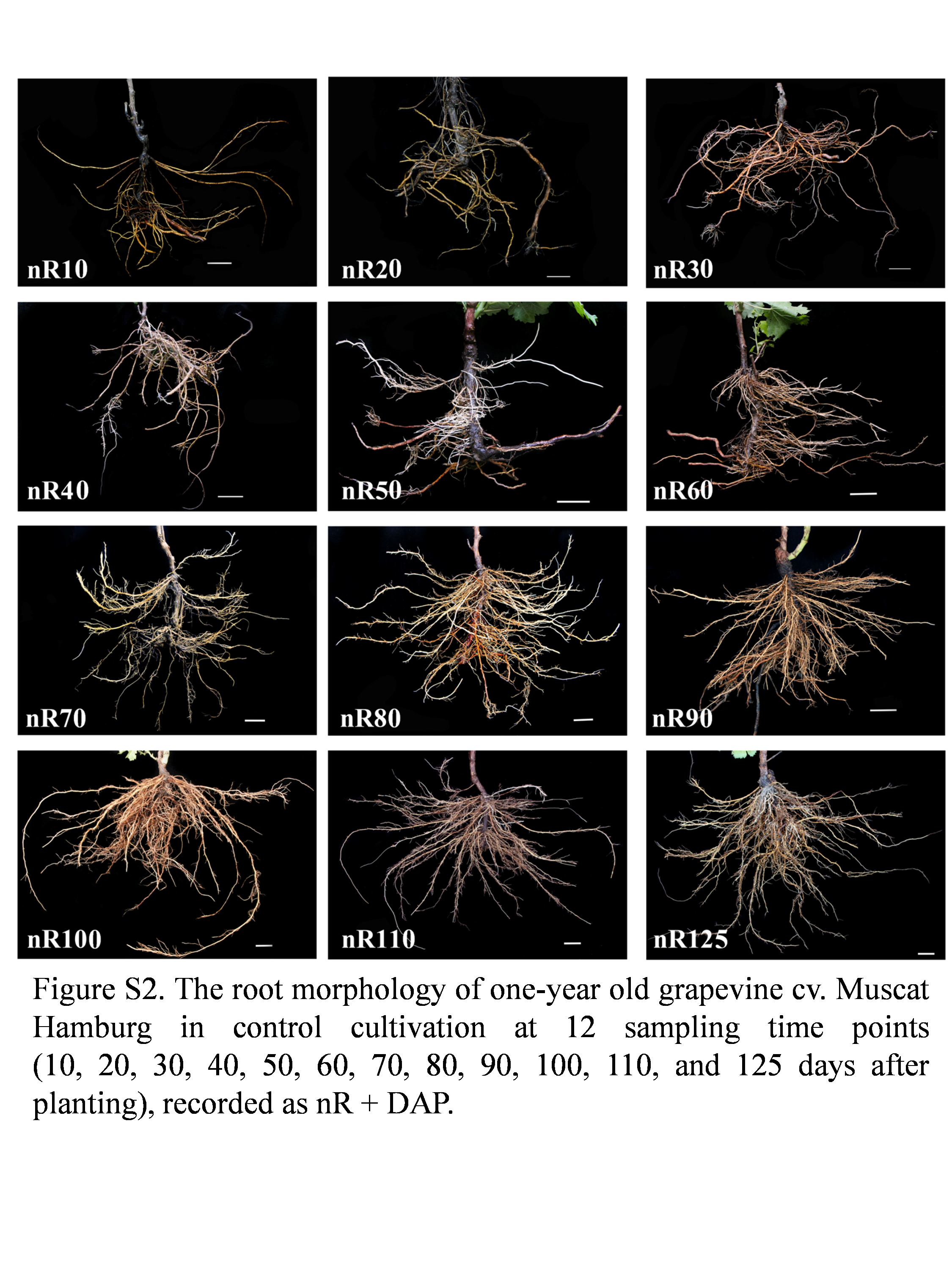

Supplement: Supplementary file 1 [file ijms-21-03513-s001.zip › Figure S2.tif]

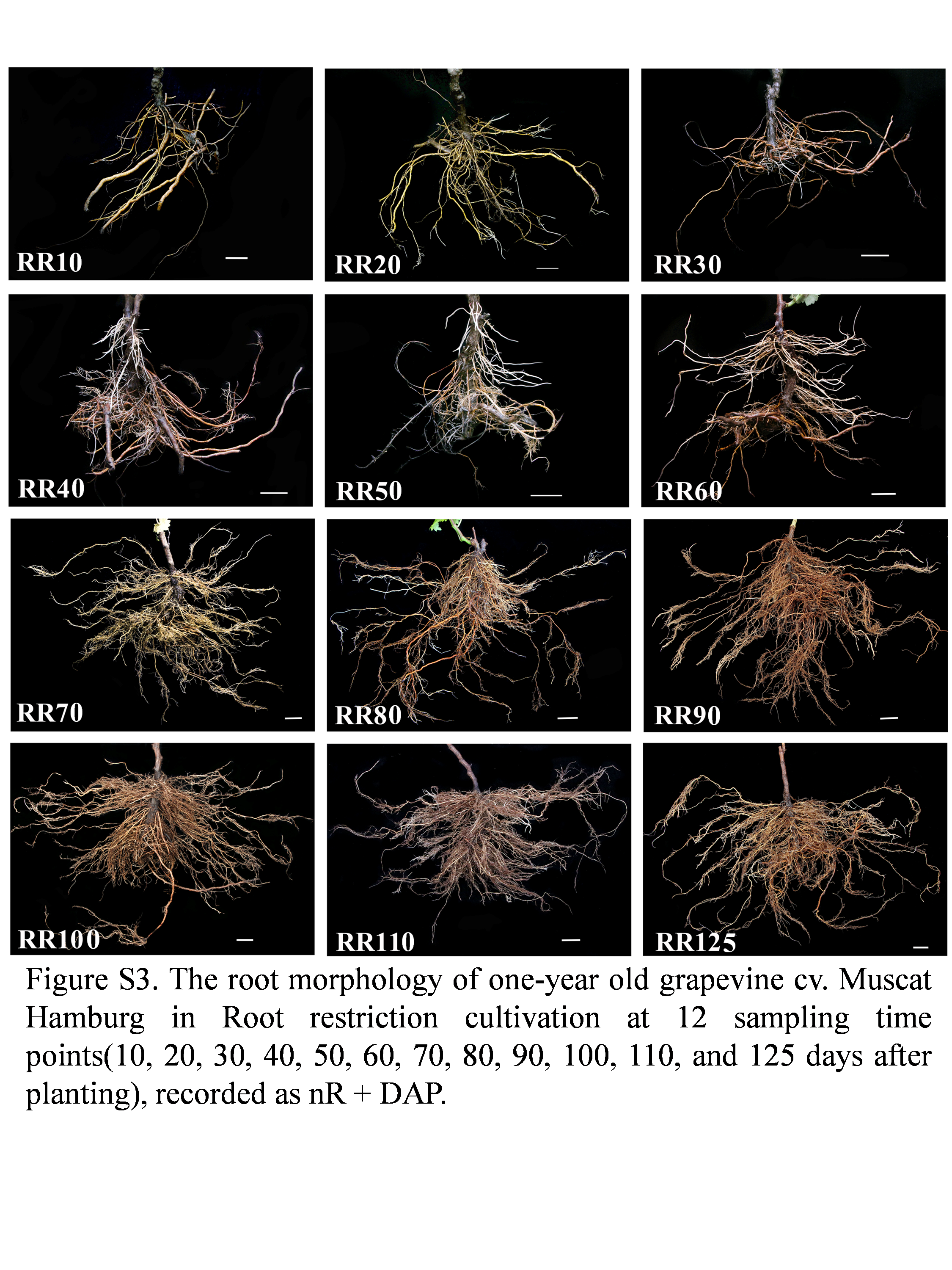

Supplement: Supplementary file 1 [file ijms-21-03513-s001.zip › Figure S3.tif]

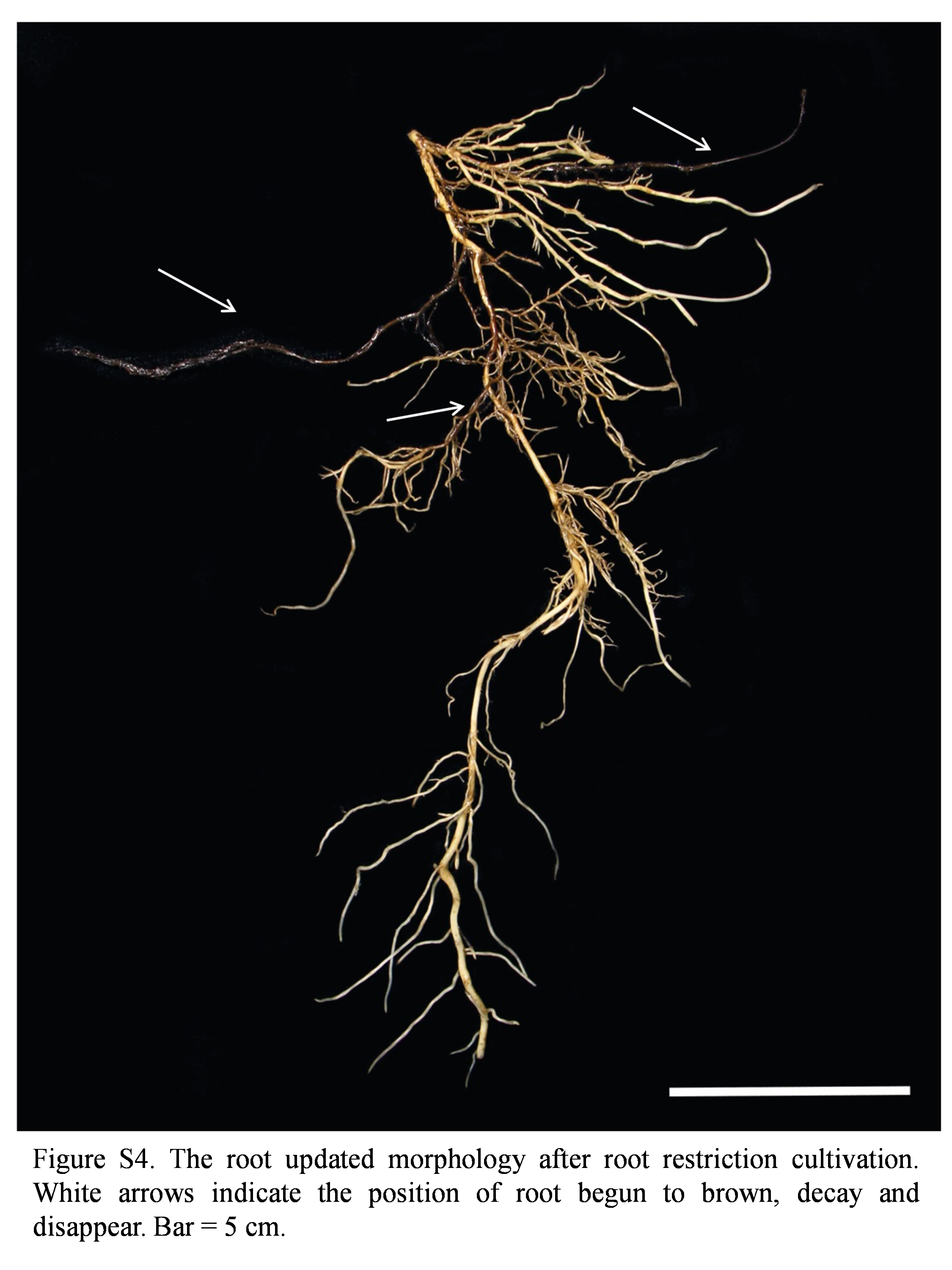

Supplement: Supplementary file 1 [file ijms-21-03513-s001.zip › Figure S4.tif]

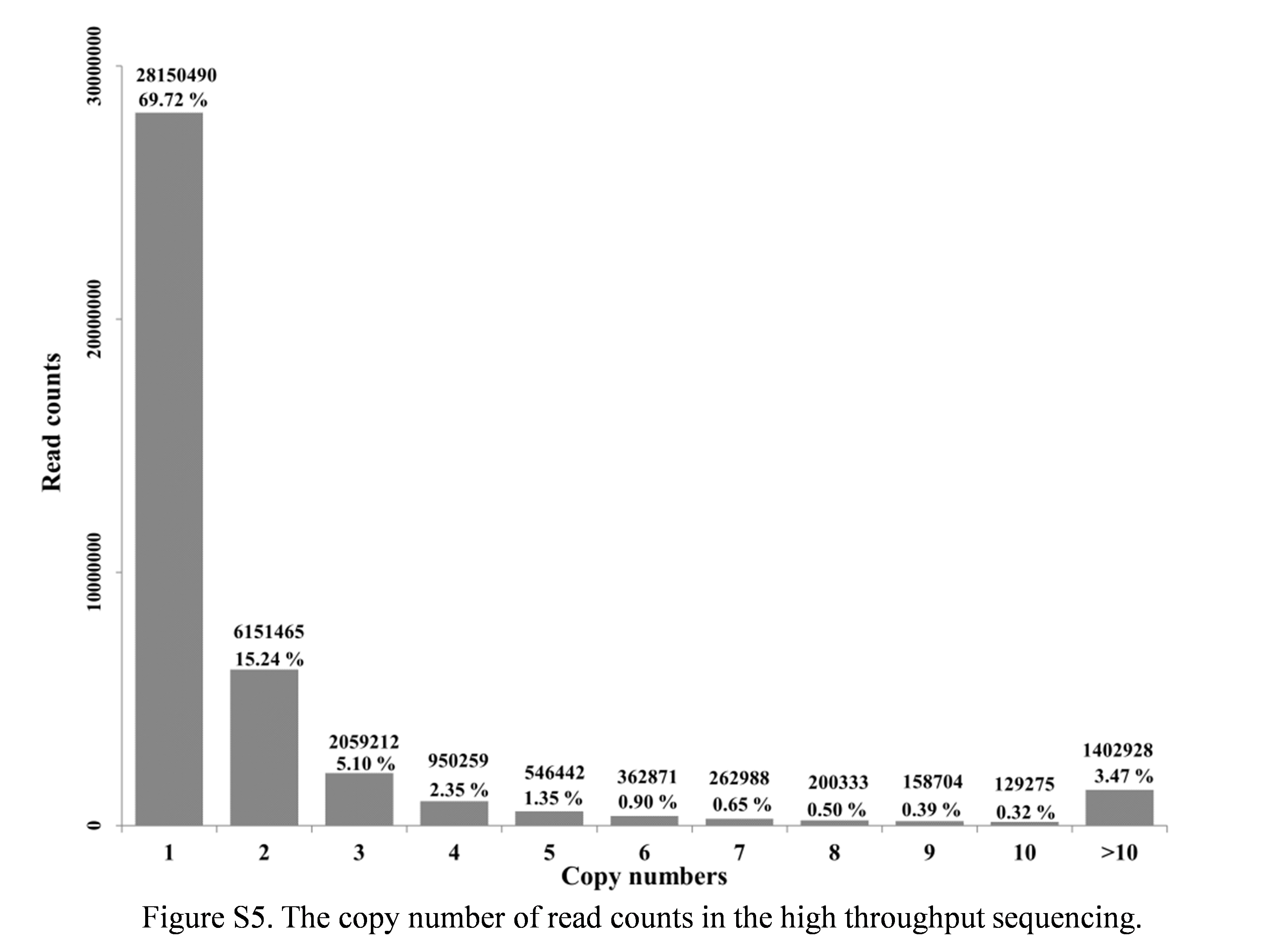

Supplement: Supplementary file 1 [file ijms-21-03513-s001.zip › Figure S5.tif]

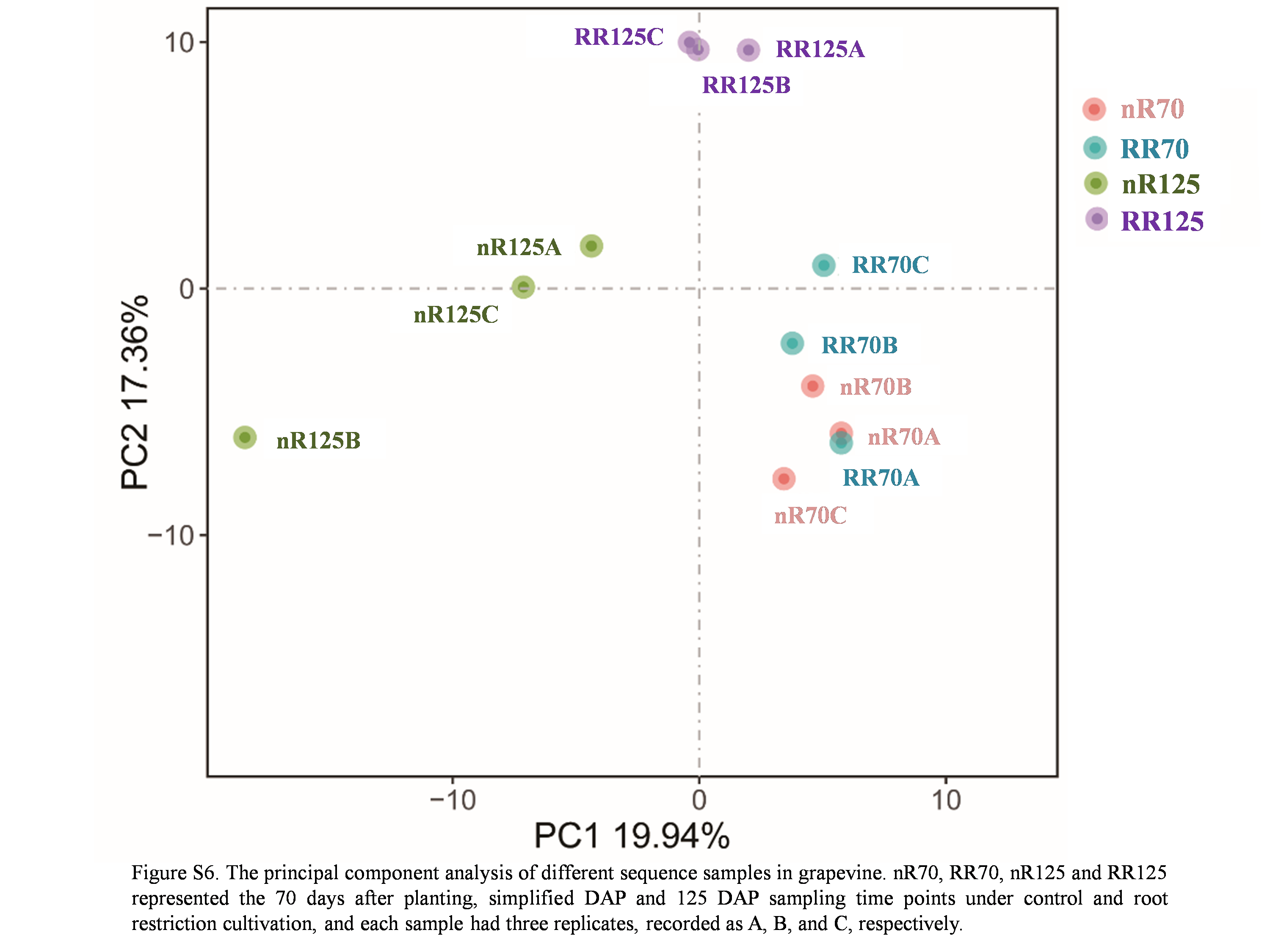

Supplement: Supplementary file 1 [file ijms-21-03513-s001.zip › Figure S6.tif]
